# Endothelin Receptor Blocker Reverses Breast Cancer–Induced Cardiac Remodeling

**DOI:** 10.1016/j.jaccao.2023.02.004

**Published:** 2023-05-02

**Authors:** Zaid H. Maayah, Mourad Ferdaoussi, Aristeidis E. Boukouris, Shingo Takahara, Subhash K. Das, Mostafa Khairy, John R. Mackey, Edith Pituskin, Gopinath Sutendra, D. Ian Paterson, Jason R.B. Dyck

**Affiliations:** a Department of Pharmaceutical Sciences, College of Pharmacy, Qatar University Health, Qatar University, Doha, Qatar; b Faculty Saint-Jean, University of Alberta, Edmonton, Alberta, Canada; c Department of Medicine, University of Alberta, Edmonton, Alberta, Canada; d Division of Cardiovascular Surgery, Tohoku University Graduate School of Medicine, Sendai, Miyagi, Japan; e Cardiovascular Research Centre, Department of Pediatrics, Faculty of Medicine and Dentistry, University of Alberta, Edmonton, Alberta, Canada; f Cross Cancer Institute, Edmonton, Alberta, Canada; g Division of Cardiology, University of Ottawa Heart Institute, University of Ottawa, Ottawa, Ontario, Canada

**Keywords:** breast tumor, cardiac remodeling, endothelin system

## Abstract

**Background:**

Although some cancer therapies have overt and/or subclinical cardiotoxic effects that increase subsequent cardiovascular risk in breast cancer patients, we have recently shown that the breast tumor itself can also induce cardiac hypertrophy through the activation of the endothelin system to contribute to cardiovascular risk. However, the extent to which the suppression of the activation of the endothelin system could improve cardiac remodeling in breast cancer patients has yet to be investigated.

**Objectives:**

We aimed to retrospectively assess the cardiac morphology/function in patients with breast cancer before receiving cancer chemotherapy and to investigate if the suppression of the activation of the endothelin system improves cardiac remodeling in a mouse model of breast cancer.

**Methods:**

Our study involved 28 previously studied women with breast cancer (including 24 after tumor resection) before receiving adjuvant therapy and 17 control healthy women. In addition, we explored how the endothelin system contributed to breast cancer–induced cardiac remodeling using a mouse model of breast cancer.

**Results:**

Our results indicate that before chemotherapy, breast cancer patients already exhibit relative cardiac remodeling and subclinical cardiac dysfunction, which was associated with the activation of the endothelin system. Importantly, our mouse data also show that the endothelin receptor blocker atrasentan significantly lessened cardiac remodeling and improved cardiac function in a preclinical model of breast cancer.

**Conclusions:**

Although our findings should be further examined in other preclinical/clinical models, our data suggest that endothelin receptor blockers may play a role in cardiac health in individuals with breast cancer. (Understanding and Treating Heart Failure With Preserved Ejection Fraction: Novel Mechanisms, Diagnostics and Potential Therapeutics [Alberta HEART]; NCT02052804 and Multidisciplinary Team Intervention in Cardio-Oncology [TITAN]; NCT01621659)

In recent decades, the rate of long-term survival after breast cancer diagnosis has improved as a result of advances in screening, early diagnosis, and systemic therapies.^1^^,^^2^ However, these patients demonstrate an increased risk of developing cardiovascular disease after their cancer treatment.^3^^,^^4^ Indeed, the increased risk of cardiovascular complications after chemotherapy has been considered as a main cause of death in this population.^5^, ^6^, ^7^, ^8^ Notably, this increase in cardiovascular morbidity and mortality has historically been attributed to the known cardiovascular toxicities of many of the most frequently used breast cancer therapies.^6^, ^7^, ^8^ However, in a recent discovery, we provided evidence that challenges this dogma and suggests that the cancer cells directly influence cardiac morphology.^9^

Given that: 1) breast cancer cells secrete endothelin(ET)-1 to stimulate tumor growth;^10^ 2) endothelin receptor type A (ET_A_R) is highly expressed in both cancer cells and cardiomyocytes;^11^, ^12^, ^13^, ^14^ 3) activation of the endothelin system is known to contribute to cardiac remodeling and heart failure pathogenesis;^11^, ^12^, ^13^, ^14^ and 4) inhibition of endothelin signaling lessens heart failure and cardiac hypertrophy,^15^, ^16^, ^17^ we postulated that shared signaling pathways perpetuate endothelin axis activation in both systems. Thus, although the activation of the endothelin system is vital for breast tumor proliferation and metastasis,^10^ we hypothesized that a tumor-mediated humoral effect is also involved in the development of cardiac remodeling in breast cancer patients. We further hypothesized that this tumor-mediated humoral factor is ET-1. Consistent with this notion, we previously reported that breast cancer cells directly signal cardiomyocytes and induce cardiac hypertrophy through the activation of the endothelin system.^9^ However, the suppression of the cancer cell–mediated activation of endothelin signaling and the subsequent effects on cardiac remodeling in patients with breast cancer have yet to be investigated.

Based on the previous information provided, we retrospectively reviewed left ventricular (LV) and right ventricular (RV) volumes, mass, and function as well as global and regional myocardial deformation on cardiac magnetic resonance (CMR) in a previously studied cohort of women with breast cancer.^9^ In addition, we investigated if suppressing the activation of the endothelin system improves cardiac remodeling or cardiac injury in a mouse model of human breast cancer.

## Methods

Additional methods are provided in the Supplemental Appendix.

### Experimental design and treatment protocol in mice

All animal procedures were approved by the University of Alberta Institutional Animal Care and Use Committee (Health Sciences 2 Committee; approval date: October 2020; number: AUP00001794), which conforms to the *Guide for the Care and Use of Laboratory Animals* published by the United States National Institutes of Health and the principles for biomedical research involving animals developed by the Council for International Organizations of Medical Sciences.

We purchased female athymic nude mice from Charles River Laboratories. We housed these mice at 25°C, 12:12-hour light/dark cycle with ad libitum access to food and water. At 18 weeks of age, all mice received subcutaneous estrogen implants (0.5 mg/pellet/mouse, 21-day release) at the left shoulder to promote the growth of the upcoming implanted breast cancer cells. Five days later, mice were injected into the subcutaneous flank with 5 × 10^6^ ZR-75-1 breast cancer cells (1:1 Matrigel [Corning] to ZR-75-1 media) (n = 20) or vehicle (n = 10). Three weeks after estrogen implantation, once tumors become visible, ZR-75-1–injected mice were then randomized to receive either vehicle (n = 10) or water containing a selective ET_A_R blocker (atrasentan^18^ [10 mg/kg/d]) (n = 10) for 4 weeks.

### Retrospective cohort analysis

We obtained ethics approval from the University of Alberta Biomedical Panel and the Alberta Cancer Board along with informed written consent from all participants. Our study includes data from 28 women with breast cancer before receiving systemic cancer treatment.^9^ We screened patients with early-stage breast cancer with planned anthracycline or trastuzumab therapy from a tertiary care cancer hospital (Cross Cancer Institute). After a comprehensive medical review, we excluded patients with diabetes, hypertension, or previous cancer therapies. Of note, 24 of our patients underwent breast cancer lumpectomy at a median of 44 days before CMR, and the remaining 4 received cancer treatment in a neoadjuvant setting. Patients receiving neoadjuvant treatment underwent CMR before receiving this therapy. Biometric data (height, weight, blood pressure, and heart rate) were recorded on the day of CMR. A cohort of age-, body mass index–, body surface area–matched healthy control women (n = 17) were included in the study (Supplemental Table 1). CMR was performed on a 1.5-T system (Aera, Siemens Healthcare). Cardiac structure and function were derived from steady-state free precession cine imaging of conventional short and long-axis views.

### Statistical analysis

We used GraphPad Prism software (version 8.01, GraphPad Software, Inc) to perform our statistical analysis. Results for human data are shown as mean ± SD, whereas our results for animal data are presented as mean ± SEM. The Kolmogorov-Smirnov test was used to assess the normality of distribution of each parameter. We used 1-way analysis of variance to compare >2 groups followed by the Tukey-Kramer post hoc test for multiple pairwise comparisons or the unpaired 2-tailed *t*-test to compare 2 groups. Pearson’s correlation coefficient was used to determine the correlations between big ET-1 levels and RV/LV parameters. A *P* value <0.05 was considered statistically significant.

## Results

### ET-1 receptor blocker improves cardiac remodeling in a mouse model of human breast cancer

**Figure 1:**
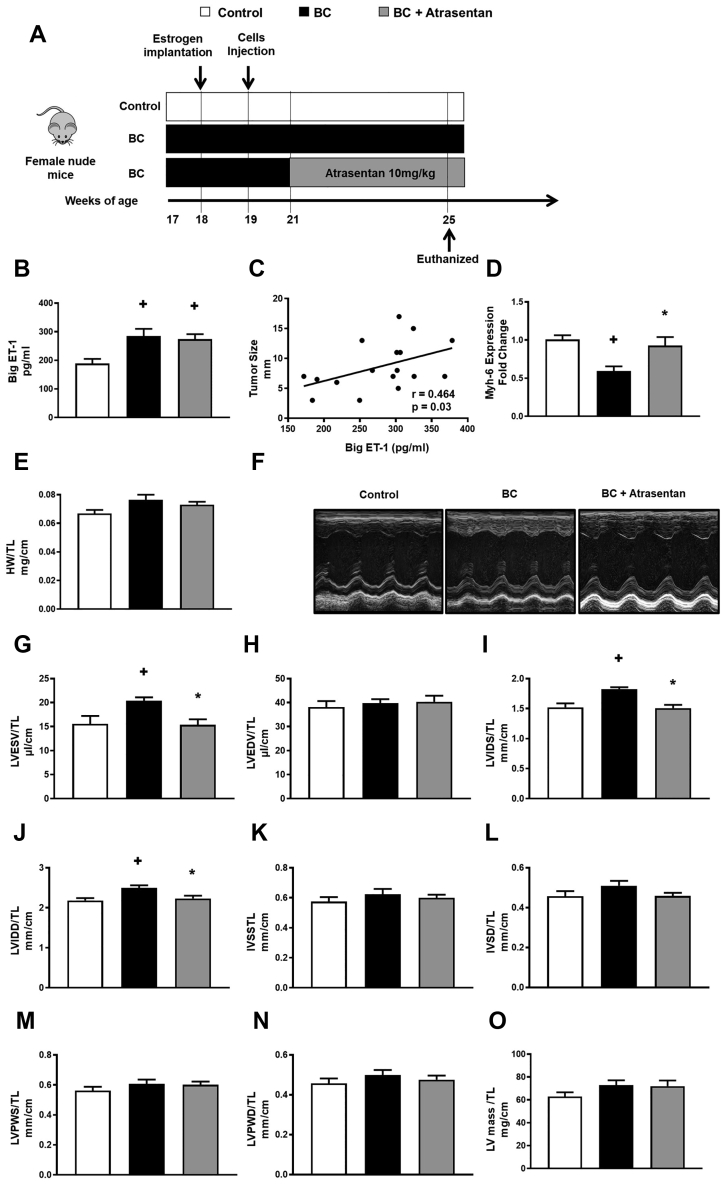
Atrasentan Improves Cardiac Remodeling in BC–Injected Mice (A) A schematic of the study design. (B) Cancer-injected mice also had high levels of big endothelin (ET)-1. (C) Big ET-1 positively correlated with tumor size. Also, there was a reduction in (D) *Myh6* level and (E) a modest increase in heart weight (HW)/tibia length (TL). However, (F) echocardiography demonstrated atrasentan improves (G) left ventricular end-systolic volume (LVESV)/TL, (H) left ventricular internal diameter systole (LVIDS) /TL, and (I) left ventricular internal diameter diastole (LVIDD)/TL in cancer-injected mice. Nevertheless, there were no significant changes in (J) left ventricular end-diastolic volume (LVEDV)/TL, (K) interventricular septum in systole (IVSS), (L) interventricular septum in diastole (IVSD), (M) left ventricular posterior wall thickness in systole (LVPWS), (N) left ventricular posterior wall thickness in diastole (LVPWD), and (O) left ventricular (LV) mass. Results are shown as mean ± SEM (n = 6-9 per group). +*P <* 0.05 vs its own control group. ∗*P <* 0.05 vs its breast cancer (BC)-injected mice.

To address our hypothesis that a tumor-mediated ET-1 effect is involved in the development of cardiac remodeling in breast cancer patients, we first set out to explore this in immunocompromised mice that had human breast cancer cells injected into their subcutaneous flank (Figure 1A, Supplemental Figure 1A) to establish tumors. As predicted, our mouse model of human breast cancer was associated with high circulating levels of big ET-1 (Figure 1B). Of interest, we observed that the circulating big ET-1 positively correlated with tumor size, suggesting that the tumor is a major contributor to the activation of the endothelin system in our mouse model of breast cancer (Figure 1C). Importantly, we found that our mouse model of breast cancer demonstrated signs of cardiac remodeling at the molecular level as evidenced by a significant reduction in *Myh6* gene expression^19^ (Figure 1D); a slight but not significant increase in heart weight/tibia length (TL); and significant increases in LV end-systolic volume/TL, LV internal diameter systole/TL, and LV internal diameter diastole/TL compared with control mice (Figures 1F, 1G, 1I, and 1J). Conversely, breast cancer–injected mice treated with the ET_A_R blocker atrasentan showed a significant reduction in LV end-systolic volume/TL, LV internal diameter diastole/TL, and LV internal diameter systole/TL (Figures 1G, 1I, and 1J) compared with breast cancer–injected mice administered vehicle. However, there were no significant changes in LV wall thickness or calculated LV mass demonstrating that breast cancer–injected mice treated with atrasentan did not display overt reduction in cardiac hypertrophy (Figures 1K to 1O). Also, our results showed that the aortic diameter and the pressure gradient across the aortic valve were unchanged in our mouse model of breast cancer and that atrasentan treatment did not alter these parameters, suggesting that the cardiac remodeling in our breast cancer model was independent from changes in blood pressure (Supplemental Figures 1B and 1C). Together, our findings indicate that the molecular signs of cardiac remodeling and differences in morphology in our breast cancer model can, at least in part, be credited to the activation of the endothelin system.

### ET-1 receptor blocker improves signs of cardiac dysfunction in a mouse model of human breast cancer

**Figure 2:**
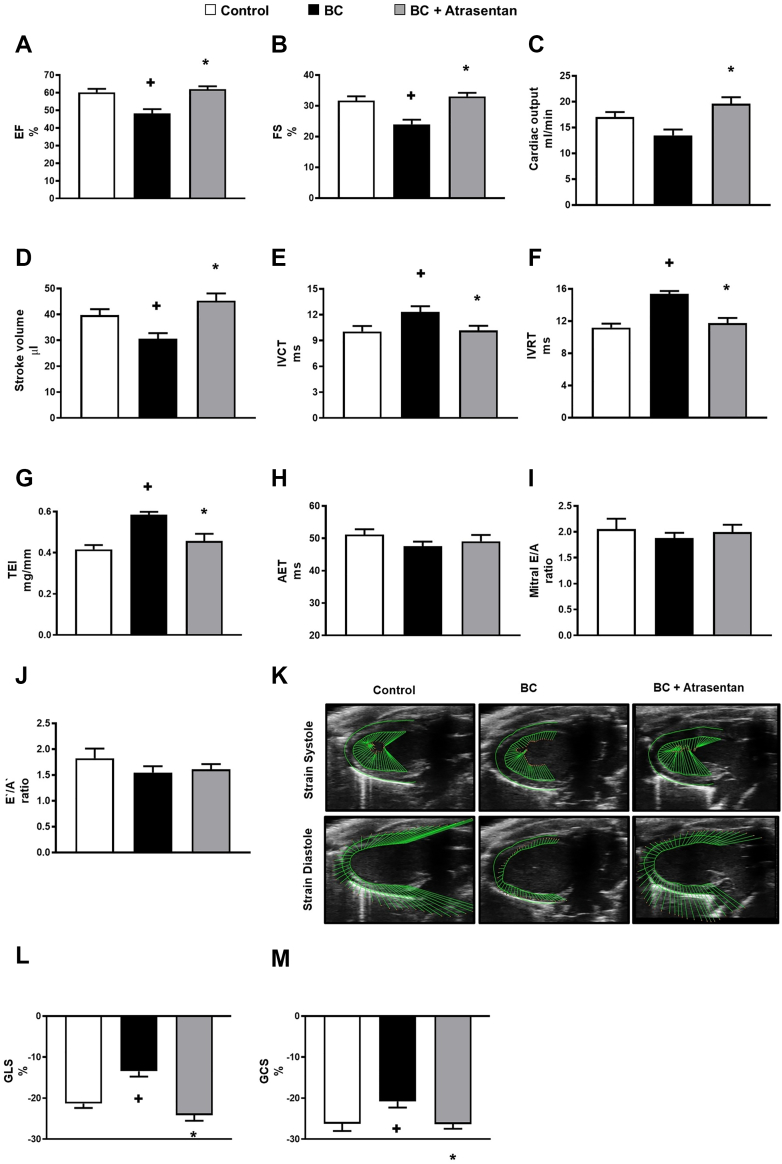
Atrasentan Improves Cardiac Dysfunction in BC-Injected Mice Four weeks after treatment of breast tumor–injected mice with vehicle or atrasentan, breast cancer (BC)-injected mice treated with atrasentan showed a significant improvement in (A) ejection fraction (EF), (B) fractional shortening (FS), (C) cardiac output, (D) stroke volume, (E) isovolumic contraction time (IVCT), (F) isovolumic relaxation time (IVRT), and (G) Tei (TEI) index. However, there were no significant changes in (H) aortic ejection time (AET), (I) mitral E-wave velocity/A-wave velocity (mitral E/A), and (J) E′/A′ in all groups. (K) Representative echocardiography of myocardial strain. Strain data demonstrated that atrasentan significantly improved (L) global longitudinal strain (GLS) and (M) global circumferential strain (GCS) in BC-injected mice. Results are shown as mean ± SEM (n = 6-9 per group). +*P <* 0.05 vs its own control group. ∗*P <* 0.05 vs its BC-injected mice treated with vehicle.

To test the hypothesis that the activation of endothelin signaling contributes to the modest cardiac dysfunction in breast cancer, we examined if atrasentan improves cardiac dysfunction in our preclinical mouse model of human breast cancer. Notably, echocardiography revealed that our mouse model of breast cancer demonstrated modest impairments in parameters of systolic function (such as ejection fraction, fractional shortening, and stroke volume) (Figures 2A to 2D) and diastolic function (such as isovolumic contraction time, isovolumic relaxation time, and Tei index) (Figures 2E to 2G). Conversely, breast cancer–injected mice treated with atrasentan showed a significant improvement in systolic and diastolic function compared with breast cancer–injected mice administered vehicle (Figure 2). In addition, we found that our mouse model of breast cancer demonstrated reduced cardiac global longitudinal strain (GLS) and global circumferential strain (GCS) compared with the controls (Figures 2K to 2M). Importantly, we observed that atrasentan significantly improved cardiac GLS and GCS in our mouse model of breast cancer compared with breast cancer–injected mice administered vehicle (Figures 2K to 2M). Collectively, our findings suggest that the cardiac dysfunction in our breast cancer model may, at least in part, be attributed to the activation of the endothelin system.

### Lack of cardiac inflammation and fibrosis in a mouse model of human breast cancer

**Figure 3:**
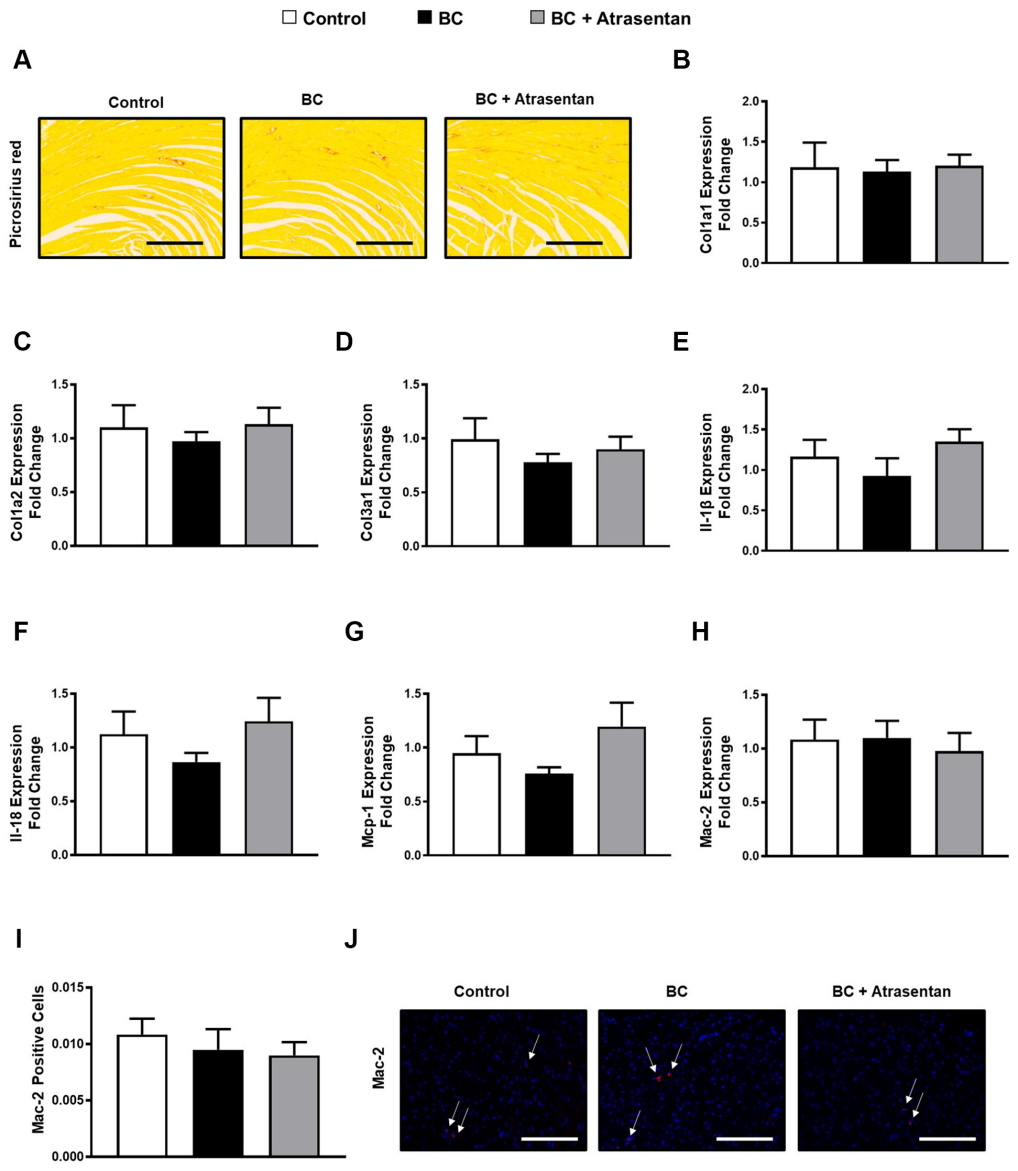
Minimal Changes in Select Inflammatory and Fibrosis Mediators in Mice (A to J) Four weeks after treatment of tumor-injected mice with vehicle or atrasentan, there were no significant changes in cardiac fibrosis/inflammation makers in all groups. (A) Representative images of mouse heart sections stained with picrosirius red. Quantification of transcript levels (B) collagen 1a1 (Col1a1), (C) collagen 1a2 (Col1a2), (D) collagen 3a1 (Col3a1), (E) interleukin (IL)-1b, (F) Il-18, (G) monocyte chemoattractant protein (Mcp)-1, and (H) Mac-2 that were normalized to Rpl32. (I) Mac-2–positive cells. (J) Representative images of mouse heart with immunostaining for Mac-2. Results for animal data are shown as mean ± SEM (n = 6-9 per group). +*P <* 0.05 vs its own control group. ∗*P <* 0.05 vs its breast cancer (BC)-injected mice.

Using picrosirius red staining of histologic cardiac sections and real-time polymerase chain reaction, we found there were no significant changes in the interstitial cardiac fibrosis (Figure 3A) or the cardiac transcript levels of fibrotic markers collagen 1a1, collagen 1a2, and collagen 3a1 (Figures 3B to 3D), respectively, in our mouse model of breast cancer. Similarly, there were no significant changes in the cardiac transcript levels of the inflammatory markers interleukin 1β, interleukin 18, monocyte chemoattractant protein-1, Galectin-3 (Mac-2) (Figures 3E to 3H), or the number of Galectin-3 (Mac-2)–positive cells (Figures 3I and 3J) in all groups of our mouse model of breast cancer. Together, our data suggest that the cardiac remodeling in our preclinical breast cancer models occurs independently from cardiac inflammation and/or fibrosis.

### Atrasentan restores endothelin-A/endothelin-B receptor expression and upregulates antioxidant markers in the heart of a mouse model of human breast cancer

**Figure 4:**
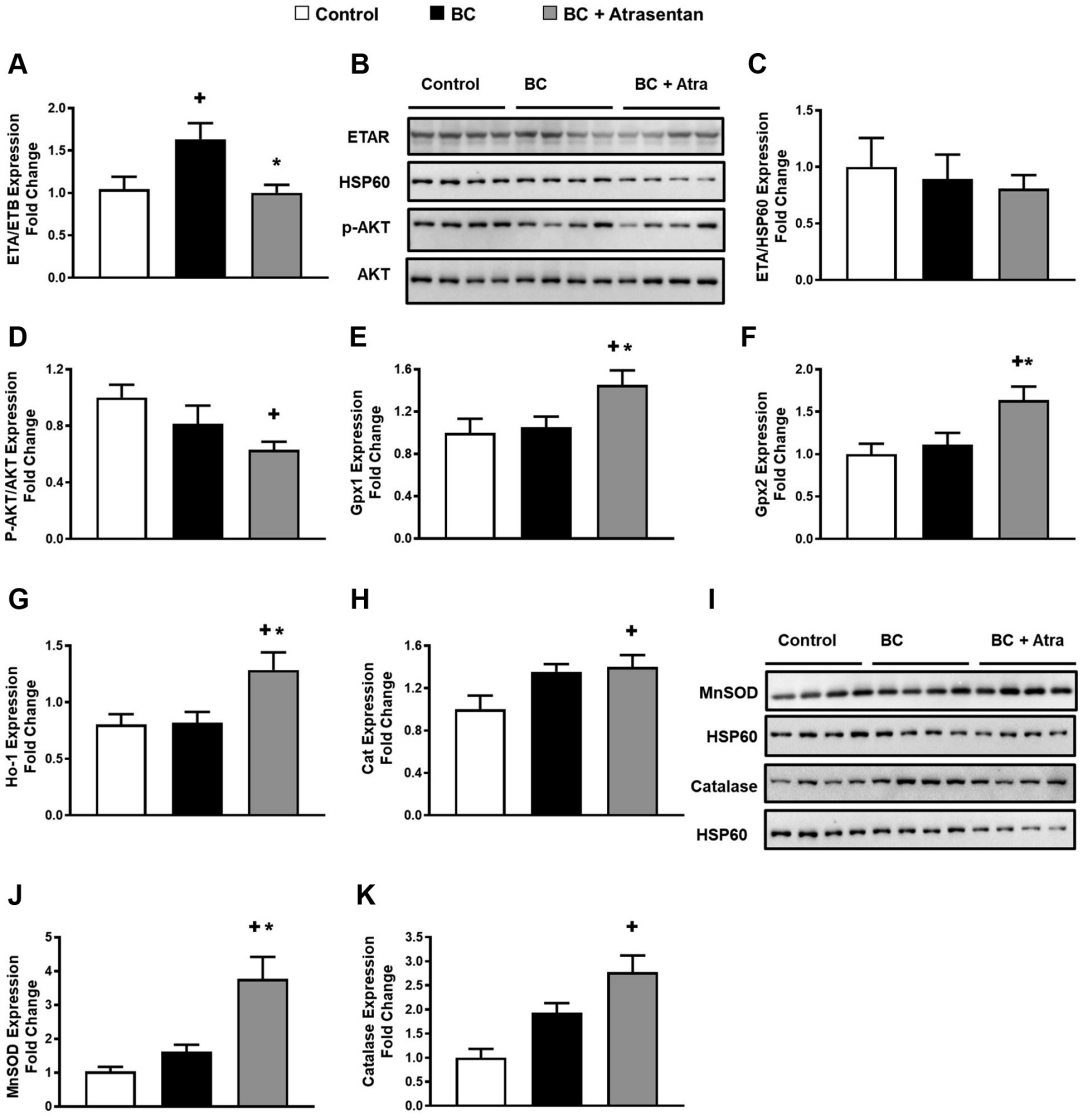
Atrasentan Downregulates p-AKT and Upregulates Antioxidants in Mice Four weeks after treatment of breast tumor–injected mice with vehicle or atrasentan, we found that atrasentan downregulated p-AKT and upregulated antioxidant markers in the heart tissue. (A) Quantification of transcript levels of endothelin(ET)-A/ETB receptor by quantitative reverse transcription polymerase chain reaction. (B) Lysates from heart were immunoblotted with antibodies against (C) ETA receptor/HSP60 and (D) p-AKT/AKT. Quantification of cardiac transcript levels of (E) glutathione peroxidase (Gpx)-1, (F) Gpx-2, (G) heme oxygenase (Ho)-1, and (H) catalase (Cat) by quantitative reverse transcription polymerase chain reaction. (I) Lysates from heart were immunoblotted with antibodies against MnSOD/HSP60 and catalase/HSP60 in the heart tissue of control and breast cancer (BC)-injected mice. Quantification of (J) MnSOD/HSP60 and (K) catalase/HSP60, respectively. Results are shown as mean ± SEM (n = 6-9 per group). +*P <* 0.05 vs its own control group. ∗*P <* 0.05 vs its BC-injected mice treated with vehicle. Some results presented in this figure have been previously reported.^9^

To test whether the cardioprotective effect of atrasentan is associated with a change in the endothelin-related pathway, we measured endothelin-A (ETA)/endothelin-B (ETB) receptor expression in the mouse heart tissues using quantitative reverse transcription polymerase chain reaction as well as the protein level of ET_A_R and phospho-AKT (p-AKT) using Western blot analysis. We found that our mouse model of breast cancer demonstrated a significant upregulation of ETA/ETB receptors compared with hearts from control mice (Figure 4A). Importantly, atrasentan significantly restored ETA/ETB receptor levels in the heart to that of the control group in our mouse model of human breast cancer (Figure 4A). However, although our mouse model of breast cancer did not demonstrate a significant change in p-AKT protein level or ETA receptor (Figures 4B and 4D), atrasentan significantly decreased p-AKT protein levels in the heart tissues (Figures 4B and 4D), suggesting that atrasentan significantly blocked the endothelin signaling in our breast cancer–injected mice. Nevertheless, the absence of elevated p-AKT in our breast cancer–injected mice administered vehicle suggested that the activation of endothelin signaling may not have occurred. Alternatively, it is possible that p-AKT was increased in hearts from vehicle-treated mice but that the levels of p-AKT normalized at the time of measurement. Because we have no direct evidence of this latter possibility, we currently cannot make any definitive conclusions about this finding.

In addition to ET receptors and p-AKT, given the antioxidant effect of ETA blockers such as atrasentan,^20^ we also measured the antioxidant effect of atrasentan in heart tissues. Notably, only breast cancer–injected mice treated with atrasentan showed a significant upregulation of the cardiac transcript and protein levels of antioxidant markers (Figures 4E to 4K). However, there were no significant changes in other oxidative stress markers in these same groups (Supplemental Figure 2), including ones that have previously been shown to be altered by atrasentan^20^ as well as ones in our previous study.^21^ Overall, our data suggest that atrasentan alters ET-1–related signaling in the hearts of breast cancer–injected mice. Thus, although we cannot explain the significant change in some antioxidant markers and the nonsignificant difference in oxidative stress markers, our data suggest that atrasentan still alters ET-1–related signaling in the hearts of breast cancer–injected mice.

### Women with breast cancer before chemotherapy display right and left cardiac remodeling relative to healthy controls

In order to translate our findings in mice to women with breast cancer, we performed a retrospective cohort study of women with mainly resected breast cancer and healthy control participants who underwent CMR scans. We have previously shown that women with breast cancer had increased LV volumes and mass with no change in left ventricular ejection fraction (LVEF) compared with the healthy control participants (Table 1).^9^ In the present study, we found that breast cancer patients also demonstrated an increase in right-sided cardiac volumes with no alteration in right ventricular ejection fraction (Table 1). Given that GLS is acknowledged as a more sensitive measure to predict subclinical cardiac events and mortality compared with LVEF,^22^^,^^23^ we also measured GLS in our women with breast cancer and the healthy controls. Compared with the healthy control women, patients with breast cancer demonstrated a nonstatistically significant reduction in LV GLS. However, a previous study showed a significant reduction in LV GLS in a larger cohort of cancer patients before receiving chemotherapy.^24^ Although women with breast cancer in our study did not demonstrate any elevation in systolic and diastolic blood pressure values at the time of baseline assessment before the CMR study, heart rate was significantly increased in breast cancer patients compared with the healthy control women (Table 1). Together, our data suggest that patients with resected breast cancer exhibit left and right-sided cardiac remodeling prior to receiving chemotherapy (Table 1).

**Table 1:** Clinical and CMR Characteristics in Patients With Breast Cancer and Healthy Control Women

	Healthy (n = 17)	Coefficient of Variations	Cancer (n = 28)	Coefficient of Variations	*P* Value
Clinical characteristics					
Age, y	59 ± 8.4	14.27	52.5 ± 10.2	19.56	0.14
Systolic blood pressure, mm Hg	125.5 ± 15.2	12.12	130 ± 15	11.55	0.36
Diastolic blood pressure, mm Hg	74.4 ± 7.7	10.45	71.5 ± 9.7	13.65	0.32
Heart rate, beats/min	68.75± 10.29	14.97	79.6 ± 13.05	16.40	0.006
Big endothelin-1, pg/mL	2.9 ± 0.47	15.88	3.4 ± 0.48	14.49	0.014
CMR characteristics					
Indexed cardiac volumes and mass					
LVEDV, mL/m^2^	73.9 ± 12.4	16.95	85.6 ± 19	22.28	0.031
LVESV, mL/m^2^	28.4 ± 4.9	17.52	33.7 ± 9.3	27.77	0.038
LV stroke volume, mL/m^2^	45.5 ± 9.3	20.51	51.9 ± 12.4	23.96	0.076
LVEF, %	61.4 ± 4.5	7.463	60.8 ± 6.4	10.53	0.73
LV mass, g/m^2^	50.7 ± 7.6	14.99	61.1 ± 9.4	15.54	<0.001
RVEDV, mL/m^2^	68.1 ± 16.7	24.63	84 ± 22.9	27.29	0.017
RVESV, mL/m^2^	28.9 ± 8.1	28.26	32 ± 11.2	35.13	0.34
RV stroke volume, mL/m^2^	40.5 ± 8.1	19.99	52.01 ± 13.9	26.75	0.027
RVEF, %	58.5 ± 6.2	10.72	62.4 ± 7.08	11.36	0.069
LAV, mL/m^2^	44.6 ± 12.5	28.11	50.9 ± 14.2	28.03	0.14
Global longitudinal strain					
LV GLS endocardial, %	−21.8 ± 2.3	10.99	−20.37 ± 2.4	11.80	0.055
LV GLS midwall, %	−21.7 ± 2.2	10.40	−20.38 ± 2.4	11.94	0.073
LV GLS epicardial, %	−18.37 ± 2.25	12.26	−17.2 ± 12.3	13.59	0.11
LV GLS average, %	−20.6 ± 2.2	10.71	−19.3 ± 2.34	12.13	0.068

Values are mean ± SD or %.

Some results presented in this Table have been previously reported.^9^

CMR = cardiac magnetic resonance; GLS = global longitudinal strain; LAV = left atrial volume; LV = left ventricular; LVEDV = left ventricular end-diastolic volume; LVEF = left ventricular ejection fraction; LVESV = left ventricular end-systolic volume; RV = right ventricular; RVEDV = right ventricular end-diastolic volume; RVEF = right ventricular ejection fraction; RVESV = right ventricular end-systolic volume.

### Relative left and right cardiac remodeling in women with breast cancer is correlated with the activation of the endothelin system

**Figure 5:**
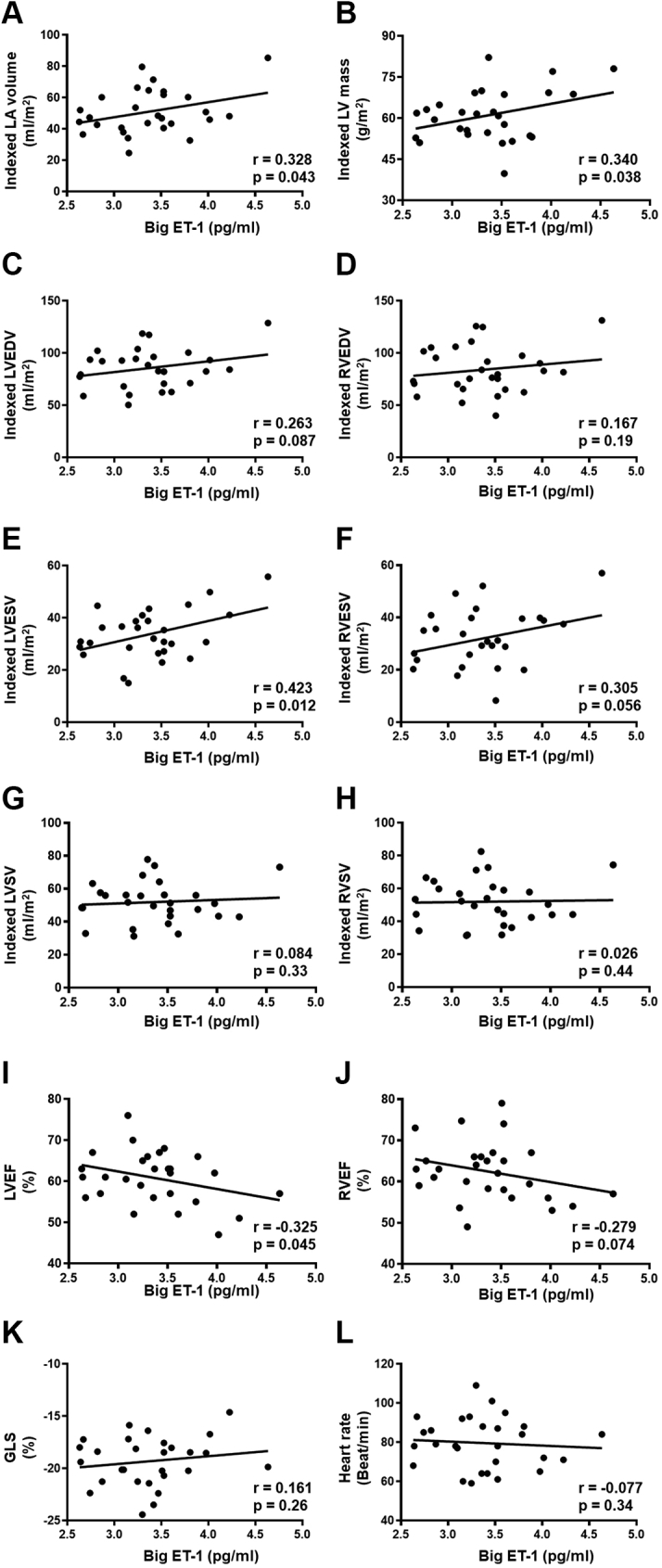
Cardiac Remodeling Is Correlated With Big ET-1 in Cancer Patients Pearson’s correlation coefficient in breast cancer patients (n = 28) revealed that (A) circulating big ET-1 positively correlated with left atrial volume (LAV) and (B) LV mass. No significant correlation between big ET-1 and (C) indexed LVEDV or (D) indexed right ventricular end-diastolic volume (RVEDV). (E) Significant positive correlation between big ET-1 concentration and indexed LVESV. No significant correlation between big ET-1 and (F) indexed right ventricular end-systolic volume (RVESV), (G) indexed left ventricular stroke volume (LVSV), or (H) indexed right ventricular systolic volume (RVSV). (I) Significant negative correlation between big ET-1 concentration and left ventricular ejection fraction (LVEF). No significant correlation between big ET-1 and (J) right ventricular ejection fraction (RVEF), (K) GLS, or (L) heart rate. Some results presented in this Figure have been previously reported.^9^ Abbreviations as in Figures 1 and 2.

Based on our previous study,^9^ the increase in LV mass and volumes in women with breast cancer was associated with an elevation in big ET-1 (Figure 5). However, whether or not RV volumes as well as left atrial volume (LAV) and GLS are correlated with the activation of the endothelin system has not been previously studied. In the current study, we found that circulating levels of big ET-1, a marker of endothelin system activation,^16^^,^^25^ correlated positively with LAV (Figure 5A). However, there was no significant correlation between big ET-1 and RV structure and function. Overall, these data suggest that the activation of the endothelin system might contribute, at least in part, to cardiac remodeling in women with breast cancer.

## Discussion

We observed that the activation of the endothelin system in women with breast cancer was correlated with left atrial remodeling. This is a potentially important finding because LAV is considered a sensitive predictor of outcomes in patients with cardiovascular disease and is an early marker of heart failure with preserved ejection fraction.^26^, ^27^, ^28^ We did not find a significant correlation between LV GLS (an emerging metric of cardiac function and prognosis) and big ET-1 within the patients with breast cancer. However, the lack of correlation in our patient cohort might be attributed to our relatively small sample size. Alternatively, the majority of our patients with breast cancer (24/28) had their tumors surgically removed before the blood draw and CMR measurements. Thus, it is possible that tumor resection lessens the production of tumor-mediated big ET-1. Nevertheless, animal data suggest that increased circulating big ET-1 contributes to subclinical cardiac dysfunction (Central illustration).

**Central Illustration:**
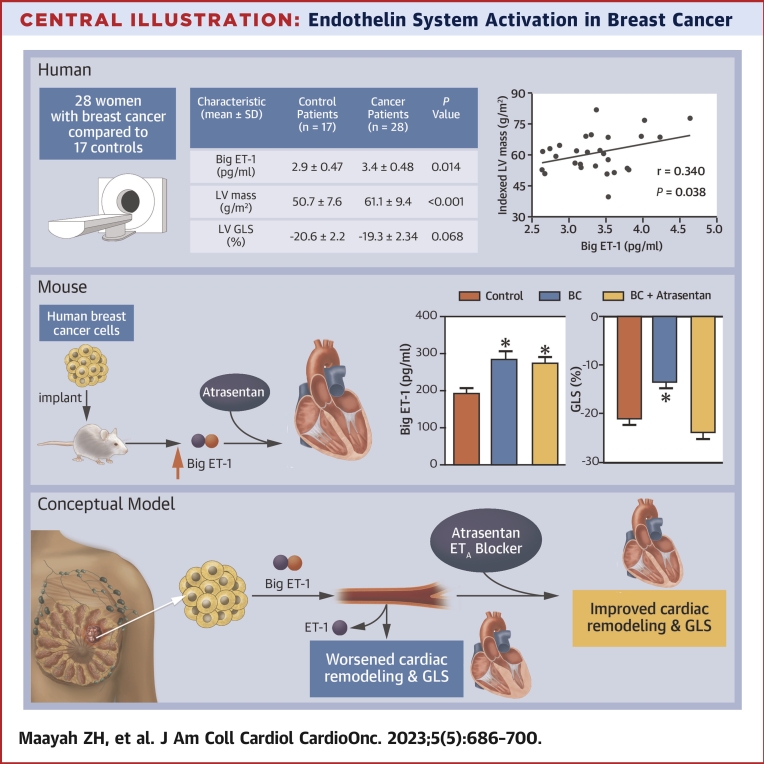
Endothelin System Activation in Breast Cancer Women with breast cancer (BC) who underwent cardiac magnetic resonance showed significant increases in circulating big endothelin-1 (ET-1) concentrations and left ventricular (LV) mass as well as a worsening in LV global longitudinal strain (GLS) compared with healthy control women. LV mass was correlated with big ET-1 concentrations in these women. Mice injected with human breast cancer cells were used to test if ET-1 receptor blockade using the ET-1 receptor blocker atrasentan improved cardiac remodeling. Similar to humans, BC was associated with a significant increase in circulating big ET-1 levels and worsening LV GLS compared with control mice. However, ET-1 receptor blockade using atrasentan prevented the worsening in LV GLS. The conceptual model suggests that ET-1 receptor blockade may be effective in improved cardiac remodeling and GLS in women with breast cancer.

We also observed that resting heart rate was significantly increased in breast cancer patients compared with the healthy control women. This observation has been reported in other studies^29^, ^30^, ^31^ but has never been mechanistically explained. It is possible that many factors can affect heart rate in cancer patients such as mental stress, anxiety, and depression.^32^ Also, although anemia could be another important factor that can increase heart rate to compensate for the decreased oxygen-carrying capacity in patients with cancer,^33^ we do not expect that this is the case in our patients because they were not anemic. Notably, the elevated heart rate in our patients might be attributed to the activation of the sympathetic nervous system, potentially by ET-1. In support of this, previous studies have shown that ET-1 stimulates stress-related sympathetic nervous system via the activation of ET_A_R.^34^^,^^35^ Indeed, stimulation of ET_A_R increases the release of norepinephrine from sympathetic neurons, resulting in the activation of cardiac β-receptors and a subsequent elevation in heart rate.^36^ However, given that most of our patients had tumor resection before the heart rate and CMI measure, it is difficult to attribute this elevation in heart rate to the production of endothelin by the primary breast cancer. Indeed, healing responses postsurgery or physiologic stress can trigger the release of circulating factors such as big ET-1 and also elevate heart rate. However, given that ET-1 (∼40 seconds) and big ET-1 (∼23 minutes) have short half-lives,^33^^,^^37^ intermittent elevations in circulating ET-1 and big ET-1 levels produced by the primary tumor or residual cancer cells might be masked and/or the time of day collection of blood may also contribute to changes in ET-1 levels.^38^ Also, it is possible that there might be upregulation of ET_A_Rs in the body, making it more susceptible to effects from endogenous ET-1 such as the sympathoexcitatory effect.^34^ These limitations of our study have yet to be fully explored.

Based on the associations identified in our human study, we attempted to explore causality in our mouse model of human breast cancer. We show that the presence of breast cancer is associated with the activation of the endothelin system and that ET-1 receptor blockade significantly attenuated cardiac remodeling in our mouse model of human breast cancer. Although other mouse models of cancer can induce cardiac cachexia, our findings indicate that the tumor itself causes a modest cardiac hypertrophy. Although the exact reasons for the different cardiac effects in other tumor-bearing mouse models is not known, the type of cancer, the time period after cardiac study, and the differing secreted factors may play a role. Nevertheless, based on our findings, we predict that the suppression of the activation of the endothelin system could improve cardiac remodeling and the subclinical cardiac dysfunction in our breast cancer patients. Thus, given the potential synergistic effect of the ETA blocker and breast cancer chemotherapies,^39^ we propose that the endothelin system may constitute an important therapeutic target to mitigate cardiovascular risk in women with breast cancer. Indeed, it has already been shown that endothelin receptor blockers such as atrasentan potentiate the antiproliferative effect of breast cancer therapies such as trastuzumab in breast cancer cells,^39^^,^^40^ and this may be worthy of further investigation.

Considering the fact that breast cancer cells release proinflammatory factors to contribute to cancer progression,^41^ it is reasonable to assume that the development of cardiac remodeling in our breast cancer model might also be attributed to inflammation and subsequent cardiac fibrosis. However, although previous work has suggested that inflammation has an important role in the pathogenesis of cancer-related heart disease,^29^ we do not expect this is the case in our study. Indeed, the cardiac remodeling in our mouse model of breast cancer lacks cardiac inflammation and/or fibrosis. Thus, it is unlikely that the cardiac remodeling associated with breast cancer is caused by inflammation and/or fibrosis. Consistent with this notion, we have previously shown that conditioned medium of human breast cancer cells that release inflammatory factors were not able to induce cardiomyocyte hypertrophy.^9^

### Study limitations

An important limitation of the current study is that most of our patients had their tumor removed surgically before the blood draw and CMR measure. Thus, it is plausible that the absence of tumor lessens the overall production of circulating big ET-1. Also, circulating big ET-1 levels might not accurately reflect the amount of big ET-1 located in the microenvironment surrounding the tumor or the heart. In addition, we cannot rule out potential confounding variables such as healing responses postsurgery or a stress-linked increase in catecholamines. Indeed, the increased sympathetic activity could elevate shear stress and thereby trigger the release of circulating factors such as big ET-1. Thus, the increased big ET-1, heart rate, and LV mass might be attributed to elevated sympathetic activity. In addition, we used a relatively small sample size of patients and healthy women in our study; thus, these findings should be confirmed in a larger number of patients with breast cancer and their matched control at baseline before the surgical removal of the tumor.

Another important limitation of the current study is that for our mouse studies, we injected cancer cells into the subcutaneous flank but not orthotopically in the mammary fat pad, which would have been more clinically appropriate. Also, although our findings indicate that the molecular signs of cardiac remodeling in our breast cancer mouse model can, at least in part, be credited to the activation of endothelin system, we did not confirm our finding using another breast cancer line to rule out cell-type specific findings. Furthermore, we did not provide a direct measurement of arterial pressure in our mouse model. Finally, the lack of significant changes in some cytokines as well as Mac-2 levels does not rule out the possibility that inflammation is occurring. This is especially true given that we used an athymic nude mouse; thus, it is difficult to predict how adaptive immunity may play a role in cardiac changes in cancer patients. Also, it is possible that inflammation preceded the cardiac changes, and they were missed in our experiments.

## Conclusions

In summary, we show that there is a significant correlation between the activation of the endothelin system and cardiac remodeling as well as cardiac dysfunction in breast cancer patients even after tumor resection. However, whether or not the activation of the endothelin system directly causes cardiac injury and remodeling in our patients is currently unknown. Nevertheless, our data indicate that ET-1 receptor blockade significantly improved cardiac remodeling in our mouse model of human breast cancer. Thus, the presented data suggest that ET-1 receptor blockade may be important for cardiac health in individuals with breast cancer, and this should be examined further in preclinical models and clinical studies. Lastly, although we use atrasentan in our mouse studies, results from the SONAR (Study of Diabetic Nephropathy with Atrasentan) study suggest worsening heart failure with atrasentan.^42^ Thus, other drugs that inhibit ET-1 signaling such as empagliflozin^43^^,^^44^ could be considered instead of atrasentan.

Perspectives **COMPETENCY IN MEDICAL KNOWLEDGE:** Although some cancer therapies have overt and/or subclinical cardiotoxic effects that increase subsequent cardiovascular risk in breast cancer patients, women with breast cancer exhibit relative cardiac remodeling and cardiac dysfunction even before chemotherapy. We show that there is a significant correlation between the activation of the endothelin system and cardiac remodeling as well as cardiac dysfunction in breast cancer patients even after tumor resection. However, whether or not the activation of the endothelin system directly causes cardiac injury and remodeling in our patients is currently unknown.

**TRANSLATIONAL OUTLOOK:** Our data indicate that ET-1 receptor blockade improved cardiac remodeling in our mouse model of human breast cancer. Thus, the presented data suggest that ET-1 receptor blockade may be important for cardiac health in individuals with breast cancer, and this should be examined further in clinical and preclinical models. If confirmed, ET-1 receptor blockers may hold promise to reduce the risk of incident heart disease in cancer survivors.

## Funding Support and Author Disclosures

This work was supported by a grant from the Canadian Institutes of Health Research and a grant from the generous supporters of the Lois Hole Hospital for Women through the Women and Children's Health Research Institute, University of Alberta to Dr Dyck. Dr Dyck is a Canada Research Chair in Molecular Medicine. All other authors have reported that they have no relationships relevant to the contents of this paper to disclose.
