# Supplementary material for: Endothelin Receptor Blocker Reverses Breast Cancer–Induced Cardiac Remodeling
Source: JACC CardioOncol. 2023 May 2;5(5):686–700. doi: 10.1016/j.jaccao.2023.02.004 (PMC10635889; doi:10.1016/j.jaccao.2023.02.004)
Supplement: Supplemental Materials [file mmc1.docx]

**SUPPLEMENTAL APPENDIX**

**Endothelin Receptor Blocker Reverses** **Breast Cancer-Induced Cardiac Remodeling**

**Materials**

ZR75-1, a human breast cancer cell line, was purchased from American Type Cell Culture ((ATCC), Manassas, VA). Estrogen pellets were obtained from Innovative Research (Innovative Research, US). Galectin-3 (Mac-2) primary antibody and TRIzol reagent were bought from Cedarlane (Cedarlane, CA) and Invitrogen (Invitrogen®, Carlsbad, CA, USA), respectively. Secondary antibody conjugated with Alexa Fluoro 594 Nuclei, human big ET-1 assay kits and ProLong Gold Antifade Mountant with DAPI were purchased from ThermoFisher (ThermoFisher; CA). Mouse big ET-1 assay kit (ABIN772876) was obtained from antibodies-online GmbH (Aachen, Germany).

**Trial registration**

ClinicalTrials.gov NCT02052804, February 3rd, 2014, for the healthy control subjects, and ClinicalTrials.gov NCT01621659, June 18th, 2012, for the breast cancer patients.

**MRI Protocol**

Both women with breast cancer and the healthy control women had a non-contrast cardiac MRI scan performed on them using a 1.5T magnet (Siemens Healthcare, Erlangen, Germany). Image acquisition and analysis was performed as described previously ^1^. A method of disks approach from a short axis stack of cines was used to calculate RV and LV volumes and mass. Finally, we used a modified area-length biplane method to estimate Left atrium volumes as described previously ^1^.

**Global Myocardial Deformation**

We used custom in-house software (MATLAB, The Mathworks, Natick, MA, USA) to analyze LV global longitudinal strain (GLS) as described previously ^1^.

**Blood Sample Collection**

At the time of MRI scan, the blood was drawn from both women with breast cancer and the healthy controls. Blood samples were then kept in 4ºC fridge and processed for storage following approval from our institutional ethics review board. Subsequent to this, we accessed the samples, spun them at 3000 rpm for 10 min at room temperature and stored the plasma at –80ºC.

**Cell Culture**

We cultured our ZR75-1 cells, a human breast cancer cell line, in 75 cm2 tissue culture flasks at 37 ◦C, 5% CO2 under a humidified environment. We used RPMI-1640 supplemented with 10% fetal bovine serum, 2mM l-glutamine, 1mM Sodium Pyruvate and 1X Antibiotic-Antimycotic ^2-4^.

**Mouse Echocardiography**

We used a Vevo 3100 high-resolution imaging system equipped with a 40-MHz transducer (Visual Sonics, Toronto, ON, Canada) to perform transthoracic echocardiography as described previously ^5,6^.

**RNA Isolation, cDNA Synthesis and Quantification of mRNA Expression by Quantitative Real-time Polymerase Chain Reaction (RT PCR)**

We used TRIzol reagent (Invitrogen®, Carlsbad, CA, USA) to isolate total RNA form 20 mg frozen heart tissue, as described previously ^7,8^. Following cDNA Synthesis, we quantified gene expression using LightCycler® 480 System (Roche Life Science), as described previously ^7,8^. The mRNA expression level of the cardiac transcripts was determined four weeks following treatment of breast tumor injected-mice with vehicle or atrasentan.

**Immunoblot analysis**

Immunoblot analysis under denaturing and reducing conditions was performed using a previously described method ^9^.

**Mouse Tissue Histology**

We used Picrosirius Red staining of heart sections to assess the interstitial cardiac fibrosis in our mouse model of human breast cancer, as described previously ^10^.

**Mouse Tissue Immunohistochemistry**

We further assessed cardiac inflammation in our mouse model of human breast cancer by measuring infiltration of macrophages into cardiac tissue using Mac-2 (also known as Galectin-3) immunofluorescence, as described previously ^11^. Paraffin embedded heart specimen was sectioned in 5 μm thickness and used for subsequent histological analyses. Sections were incubated with a primary antibody directed against Mac-2 (Galectin-3) (1:1000; Cedarlane) followed by the appropriate secondary antibody conjugated with Alexa Fluoro 594 (1:750; ThermoFisher). Nuclei were stained with DAPI using ProLong Gold Antifade Mountant with DAPI (ThermoFisher). Images from randomly selected fields of the heart were taken.

**Big ET-1 Assays**

Big ET-1 concentrations in human plasma and in our breast cancer mouse model were determined by immunoassay from ThermoFisher (BMS2266) and antibodies-online GmbH (ABIN772876), respectively.

**Supplemental Figure 1**

Five days following estrogen implants, mice were injected with breast cancer cells or vehicle. Three weeks following estrogen implantation, breast cancer cells injected mice were then randomized to receive either vehicle or water-containing a selective ETA receptor blocker, atrasentan, for 4 weeks. A: the tumor growth curves three, five and seven weeks following the injection of breast cancer cells. Four weeks following treatment of breast tumor injected-mice with vehicle or water-containing atrasentan, we found that B: aortic diameter or C: aortic pressure gradient across the aortic valve were unchanged in our mouse model of breast cancer as well as in response to atrasentan treatment. Results are shown as means ± SEM (n = 6-9 per group). + p<0.05 vs its own control group. * p<0.05 vs its breast cancer-injected mice treated with vehicle (BC).


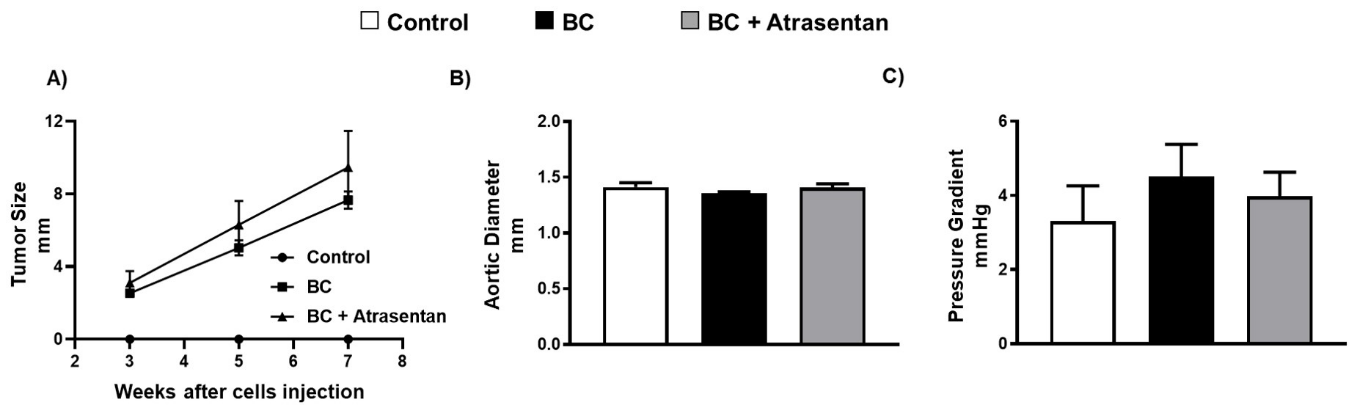


**Supplemental Figure 2**

Four weeks following treatment of breast tumor injected-mice with vehicle or atrasentan, mRNA expression levels of Nitric oxide synthase (Nos2), Nos3, Superoxide dismutase1 (Sod1), Sod2, Bnip3, and Thioredoxin-interacting protein (Txnip), were determined by qRT-PCR. We found that neither our mouse model of breast cancer nor atrasentan alter the expression level of A: Nos2, B: Nos3, C: Sod1, D: Sod2, E: Binp3, and F: Txnip in heart tissues. Results are shown as means ± SEM (n = 6-9 per group). + p<0.05 vs its own control group. * p<0.05 vs its breast cancer-injected mice treated with vehicle (BC).


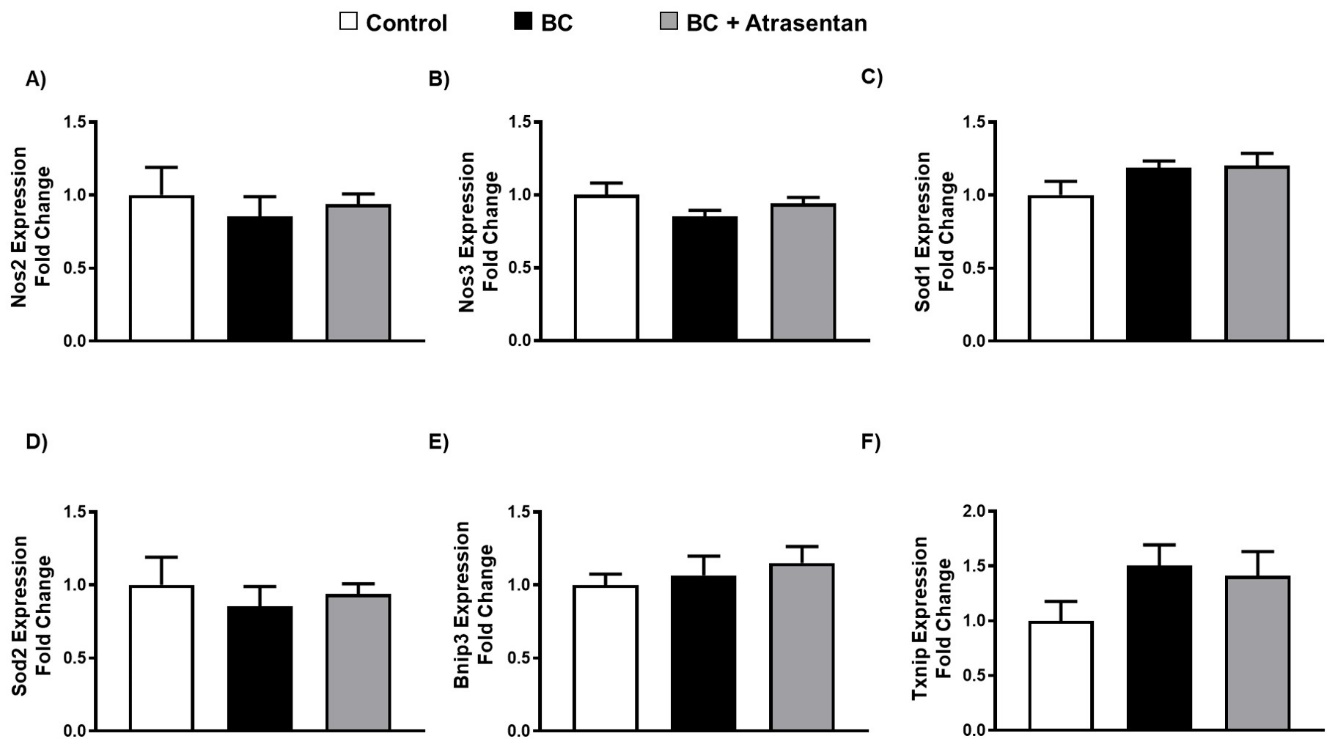


**Supplementary Table 1**

| **Characteristics (number, %)** | **Descriptive statistics (n = 28**) |
| --- | --- |
|  |  |
| Female sex | 28 (100%) |
| Hypertension (%) | 0 (0%) |
| Diabetes (%) | 0 (0%) |
|  |  |
| Cancer characteristics |  |
| Cancer type |  |
| Breast cancer | 28 (100%) |
| HER2 receptor |  |
| Negative | 19 (68%) |
| Positive | 9 (32%) |
| ER/PR receptor |  |
| Negative | 2 (7%) |
| Positive | 26 (93%) |
| Triple negative | 1 (4%) |
| Cancer Stage |  |
| 1 | 12 (43%) |
| 2 | 13 (46%) |
| 3 | 3 (11%) |
| Pre MRI lumpectomy | 24 (86%) |
|  |  |

**References**

1. Kirkham AA, Pituskin E, Thompson RB, et al. Cardiac and cardiometabolic phenotyping of trastuzumab-mediated cardiotoxicity: a secondary analysis of the MANTICORE trial. *Eur Heart J Cardiovasc Pharmacother.* 2022;8(2):130-139.

2. Mahrouf-Yorgov M, Augeul L, Da Silva CC, et al. Mesenchymal stem cells sense mitochondria released from damaged cells as danger signals to activate their rescue properties. *Cell death and differentiation.* 2017;24(7):1224-1238.

3. Kobashigawa LC, Xu YC, Padbury JF, Tseng YT, Yano N. Metformin protects cardiomyocyte from doxorubicin induced cytotoxicity through an AMP-activated protein kinase dependent signaling pathway: an in vitro study. *PloS one.* 2014;9(8):e104888.

4. Conley SJ, Bosco EE, Tice DA, Hollingsworth RE, Herbst R, Xiao Z. HER2 drives Mucin-like 1 to control proliferation in breast cancer cells. *Oncogene.* 2016;35(32):4225-4234.

5. Maayah ZH, Alam AS, Takahara S, et al. Resveratrol reduces cardiac NLRP3-inflammasome activation and systemic inflammation to lessen doxorubicin-induced cardiotoxicity in juvenile mice. *FEBS Lett.* 2021;595(12):1681-1695.

6. Takahara S, Ferdaoussi M, Srnic N, et al. Inhibition of ATGL in adipose tissue ameliorates isoproterenol-induced cardiac remodeling by reducing adipose tissue inflammation. *Am J Physiol Heart Circ Physiol.* 2021;320(1):H432-H446.

7. Maayah ZH, Levasseur J, Siva Piragasam R, et al. 2-Methoxyestradiol protects against pressure overload-induced left ventricular hypertrophy. *Scientific reports.* 2018;8(1):2780.

8. Maayah ZH, Althurwi HN, Abdelhamid G, Lesyk G, Jurasz P, El-Kadi AO. CYP1B1 inhibition attenuates doxorubicin-induced cardiotoxicity through a mid-chain HETEs-dependent mechanism. *Pharmacological research.* 2016.

9. Sambrook J, Fritsch EF, Maniatatis T. In: Ford, N. (Ed.), Molecular Cloning. A Laboratory Manual. Cold Spring Harbour Laboratory Press, Plainview, NY. 1989.

10. Matsumura N, Zordoky BN, Robertson IM, et al. Co-administration of resveratrol with doxorubicin in young mice attenuates detrimental late-occurring cardiovascular changes. *Cardiovascular research.* 2018;114(10):1350-1359.

11. Maayah ZH, Ferdaoussi M, Takahara S, Soni S, Dyck JRB. Empagliflozin suppresses inflammation and protects against acute septic renal injury. *Inflammopharmacology.* 2021;29(1):269-279.
